# Development of a radiolabeled caninized anti-EGFR antibody for comparative oncology trials

**DOI:** 10.18632/oncotarget.20914

**Published:** 2017-09-15

**Authors:** Judit Fazekas-Singer, Neydher Berroterán-Infante, Christina Rami-Mark, Monika Dumanic, Miroslawa Matz, Michael Willmann, Fritz Andreae, Josef Singer, Wolfgang Wadsak, Markus Mitterhauser, Erika Jensen-Jarolim

**Affiliations:** ^1^ Comparative Medicine, The Interuniversity Messerli Research Institute of the University of Veterinary Medicine Vienna, Medical University Vienna, and University of Vienna, Vienna, Austria; ^2^ Institute of Pathophysiology and Allergy Research, Center of Pathophysiology, Infectiology and Immunology, Medical University of Vienna, Vienna, Austria; ^3^ Department of Biomedical Imaging and Image-Guided Therapy, Division of Nuclear Medicine, Medical University of Vienna, Vienna, Austria; ^4^ Institute of Inorganic Chemistry, University of Vienna, Vienna, Austria; ^5^ Department for Companion Animals and Horses, University of Veterinary Medicine Vienna, Vienna, Austria; ^6^ piCHEM Forschungs- und Entwicklungs GmbH, Graz, Austria; ^7^ Department of Internal Medicine II, University Hospital Krems, Karl Landsteiner University of Health Sciences, Krems an der Donau, Austria; ^8^ CBmed GmbH-Center for Biomarker Research in Medicine, Graz, Austria; ^9^ LBI Applied Diagnostics, Vienna, Austria

**Keywords:** epidermal growth factor receptor, radio-immunotherapy, canine mammary carcinoma, canine antibody, comparative oncology

## Abstract

Due to large homology of human and canine EGFR, dogs suffering from spontaneous EGFR+ cancer can be considered as ideal translational models. Thereby, novel immunotherapeutic compounds can be developed for both human and veterinary patients. This study describes the radiolabeling of a canine anti-EGFR IgG antibody (can225IgG) with potential diagnostic and therapeutic value in comparative clinical settings. Can225IgG was functionalized with DTPA for subsequent chelation with the radionuclide ^99m^Tc. Successful coupling of 10 DTPA molecules per antibody on average was proven by significant mass increase in MALDI-TOF spectroscopy, gel electrophoresis and immunoblots. Following functionalization and radiolabeling, ^99m^Tc-DTPA-can225IgG fully retained its binding capacity towards human and canine EGFR in flow cytometry, immuno- and radioblots, and autoradiography. The affinity of radiolabeled can225IgG was determined to K_D_ 0.8 ±0.0031 nM in a real-time kinetics assay on canine carcinoma cells by a competition binding technique. Stability tests of the radiolabeled compound identified TRIS buffered saline as the ideal formulation for short-term storage with 87.11 ±6.04% intact compound being still detected 60 minutes post radiolabeling. High stability, specificity and EGFR binding affinity pinpoint towards ^99m^Tc-radiolabeled can225IgG antibody as an ideal lead compound for the first proof-of-concept diagnostic and therapeutic applications in canine cancer patients.

## INTRODUCTION

Detection of primary and secondary malignant lesions in clinical oncology currently largely depends on tracking of the first steps in glycolysis in metabolically active tissue via 2-deoxy-2-[^18^F]fluoro-D-glucose ([^18^F]FDG) in PET or PET/CT [1, 2]. Under certain circumstances, this method can display false positive results, e.g. in inflamed tissue [3] or highly metabolizing healthy tissues like brain or liver [4].

Therefore, tumor specific detection with monoclonal antibodies targeting tumor-associated antigens (TAAs) offers an ideal, refined target for novel therapeutic and diagnostic (=theranostic) strategies. One of the most thoroughly investigated TAAs is the epidermal growth factor receptor-1 (EGFR). EGFR-overexpression is reported amongst others in colorectal cancer or carcinomas of the head and neck region and highly successfully targeted by specific immunotherapy with the monoclonal antibody cetuximab [5]. Besides the diagnostic potency of radiolabeled antibodies, their therapeutic value in cancer has been recognized under the concept of theranostics [6].

Mouse models often lack important features of cancer such as genomic instability or tumor heterogeneity and do not ideally mimic human pharmacokinetics, which may cause failure in subsequent clinical trials [7]. To overcome this problem, this study relies on the concept of “comparative oncology” in order to gain more reliable pre-clinical data for human studies from real life cancer in pets. Of equal importance, comparative oncology aims to simultaneously develop active compounds for veterinary oncology and to test these compounds in veterinary clinical trials [8] which, interestingly, fall under directives for animal experiments in the EU as well as in the US [9].

To overcome these issues, dog models offer a new perspective for cancer therapy development. Moreover, dogs suffer from cancer with similar incidence rates as humans, share the same risk factors as their owners and have a strikingly similar immune system, thus, providing suitable models for human malignant diseases [10, 11]. On the other hand, diagnostic and therapeutic options are currently still very limited for animal patients and thus there is an urgent need for the development of species-specific targeted therapies.

While some canine TAAs as CEA substantially differ from the human counterpart [12], members of the EGFR family share an outstanding homology of up to 95% [13]. Therefore, the canine EGFR, expressed by canine cancer cells, could be targeted by cetuximab which led to tumor growth inhibition, similarly to the effects observed on human cancer cells [13].

Nevertheless, dog tumor patients cannot be treated with cetuximab [14, 15] as this human-mouse chimeric antibody would be regarded as foreign by the canine immune system and lead to specific hypersensitivity. This prompted us previously to generate a caninized version of cetuximab by fusing the variable regions of its murine precursor to canine constant gamma-regions [16]. Like cetuximab, this newly generated antibody “can225IgG” elicited tumor cell growth inhibition *in vitro*. Moreover, via its constant domain, can225IgG could bind to Fcγ-receptors of canine immune cells and thereby mediate antibody-dependent cellular cytotoxicity (ADCC) and phagocytosis (ADCP) of EGFR+ tumor cells [16].

This antibody served as lead compound in the present study, however, lacking a binding residue for a radiometal for diagnostic use. Therefore, a modification was done by functionalization of the multiple lysine residues with diethylene-triamine-pentaacetic acid (DTPA), to enable a subsequent radio-labeling with the metastable form of technetium-99 (^99m^Tc) for visualization in single photon emission computed tomography (SPECT). Although there are human clinical trials ongoing on tumor imaging using anti-EGFR-antibodies labeled with diagnostic nuclides ([^89^Zr]Zr-cetuximab: NCT00691548 (completed), NCT01691391 (recruiting), [^68^Ga]Ga-EGFR-affibody: NCT02916329 (recruiting), [^125^I]I-MAB425: NCT01317888 (status unknown)) [17], none of these antibodies is FDA-approved yet, nor are they exploiting the full potency of radiolabeled antibodies in terms of *therapeutic* nuclides. Therefore, by testing the stability and specificity of this newly labeled ^99m^Tc-DTPA-can225IgG construct *in vitro*, our study could contribute to the establishment of new imaging options in veterinary clinical oncology by introducing highly specific monoclonal antibodies as novel tracers for diagnosis in veterinary malignant diseases. In the specific case of canine osteosarcoma for instance, the early detection of distant metastases by a radiolabeled anti-EGFR antibody may considerably influence and improve therapeutic decisions. Furthermore, successfully applied diagnostic monoclonal antibodies could also be labeled with therapeutic radionuclides and thus represent a novel category of therapeutic options in veterinary oncology.

## RESULTS

### DTPA functionalization of can225IgG

**Figure 1:**
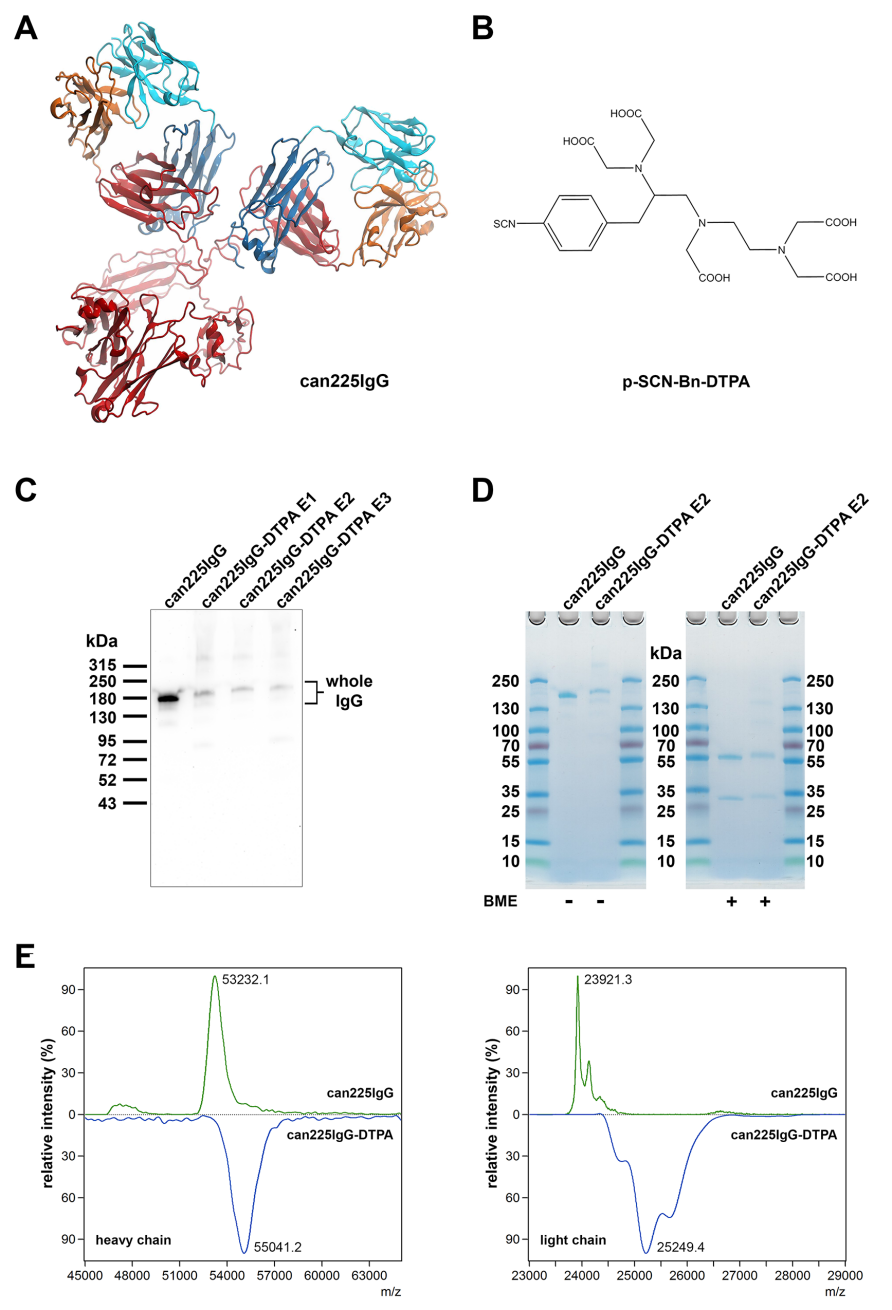
Functionalization of can225IgG with DTPA **(A)** *In silico* model of can225IgG (Model generation described in ref. 12). **(B)** Molecular structure of p-SCN-Bn-DTPA used for the functionalization of can225IgG. **(C)** Western blot with unmodified (lane 1) and DTPA-conjugated can225IgG variants E1-E3 (lanes 2-4) loaded as samples and detected with anti-dog IgG HRP. **(D)** SDS-PAGE with unmodified (lane 2) and DTPA-conjugated can225IgG E2 (lane 3) visualizing mass increase of the whole antibody (left panel) and both heavy and light chains under reducing conditions (right panel) upon functionalization. BME – 2-mercaptoethanol. **(E)** MALDI-TOF mass spectra of DTT-treated unmodified can225IgG (upper panels) and DTPA-conjugated can225IgG E2 (lower panels)

The dog-mouse chimeric anti-EGFR antibody can225IgG (Figure 1A) was functionalized with S-2-(4-isothiocyanatobenzyl)-diethylene-triamine-pentaacetic acid (p-SCN-Bn-DTPA, in following ‘DTPA’; Figure 1B) for subsequent ^99m^Tc-radiolabeling. DTPA can be covalently coupled to potentially all lysine residues of the monoclonal antibody. The heavy chain of can225IgG comprises 24, the light chain 9 lysine residues (Table 1). This results in 66 potential functionalization sites per antibody molecule. Nevertheless, not each lysine is functionalized due to sterical hindrance. In order to determine the optimal reaction conditions, 3 different experimental settings (E1-3) were tested. DTPA-can225IgG E1 was labeled with 25x molar excess of DTPA, E2 and E3 with 50x molar excess, but at different pH levels. All three experimental reaction conditions resulted in a molecular weight gain of the antibody (Figure 1C). Conditions E2 and E3 displayed more weight increase compared to E1. As in condition E3 more degraded bands were visible too, condition E2 was regarded best and thus chosen for subsequent experiments.

**Table 1:** Potential functionalization sites of can225IgG

Chain	Lysine residues	Amino acid sequence
**can225 gamma**	**24**	**QVQLKQSGPGLVQPSQSLSITCTVSGFSLTNYGVHWVRQSPGKG** **LEWLGVIWSGGNTDYNTPFTSRLSINKDNSKSQVFFKMNSLQSN** **DTAIYYCARALTYYDYEFAYWGQGTLVTVSAA**STTAPSVFPLAP SCGSQSGSTVALACLVSGYIPEPVTVSWNSGSLTSGVHTFPSIL QSSGLYSLSSMVTVPSSRWPSETFTCNVAHPATNT**K**VD**K**PVV**K**E CEC**K**CNCNNCPCPGCGLLGGPSVFIFPP**K**P**K**DILVTARTPTVTC VVVDLDPENPEVQISWFVDS**K**QVQTANTQPREEQSNGTYRVVSV LPIGHQDWLSG**K**QF**K**C**K**VNN**K**ALPSPIEEIIS**K**TPGQAHQPNVY VLPPSRDEMS**K**NTVTLTCLV**K**DFFPPEIDVEWQSNGQQEPES**K**YRMTP PQLDEDGSYFLYS**K**LSVD**K**SRWQRGDTFICAVMHEALHNH YTQ**K**SLSHSPG**K**
**can225 kappa**	**9**	**DILLTQSPVILSVSPGERVSFSCRASQSIGTNIHWYQQRTNGS** **PRLLIKYASESISGIPSRFSGSGSGTDFTLSINSVESEDIADY** **YCQQNNNWPTTFGAGTKLELKRTVA**APAVYLFQPSPDQLHTGS ASVVCLLNSFYP**K**DINV**K**W**K**VDGVIQDTGIQESVTEQD**K**DSTY SLSSTLTMSSTEYLSHELYSCEITH**K**SLPSTLI**K**SFQRSECQRVD

Lysine residues (K) are highlighted in red; amino acids in bold format constitute the variable region of the respective antibody chain.

Both heavy and light chains of DTPA-can225IgG E2 were functionalized with DTPA, determined by polyacrylamide gel electrophoresis (PAGE, see Figure 1D) and matrix-assisted laser desorption ionization - time of flight (MALDI-TOF) mass spectrometry (Figure 1E). The average mass increase (calculated from the major peaks of the heavy and light chains) was 1809 Da for the heavy and 1328 Da for the light chain, accounting for approximately 10 DTPA molecules (FW: 649.9 g/mol) per antibody.

### Specificity assessment after functionalization and ^99m^Tc radiolabeling of can225IgG

**Figure 2:**
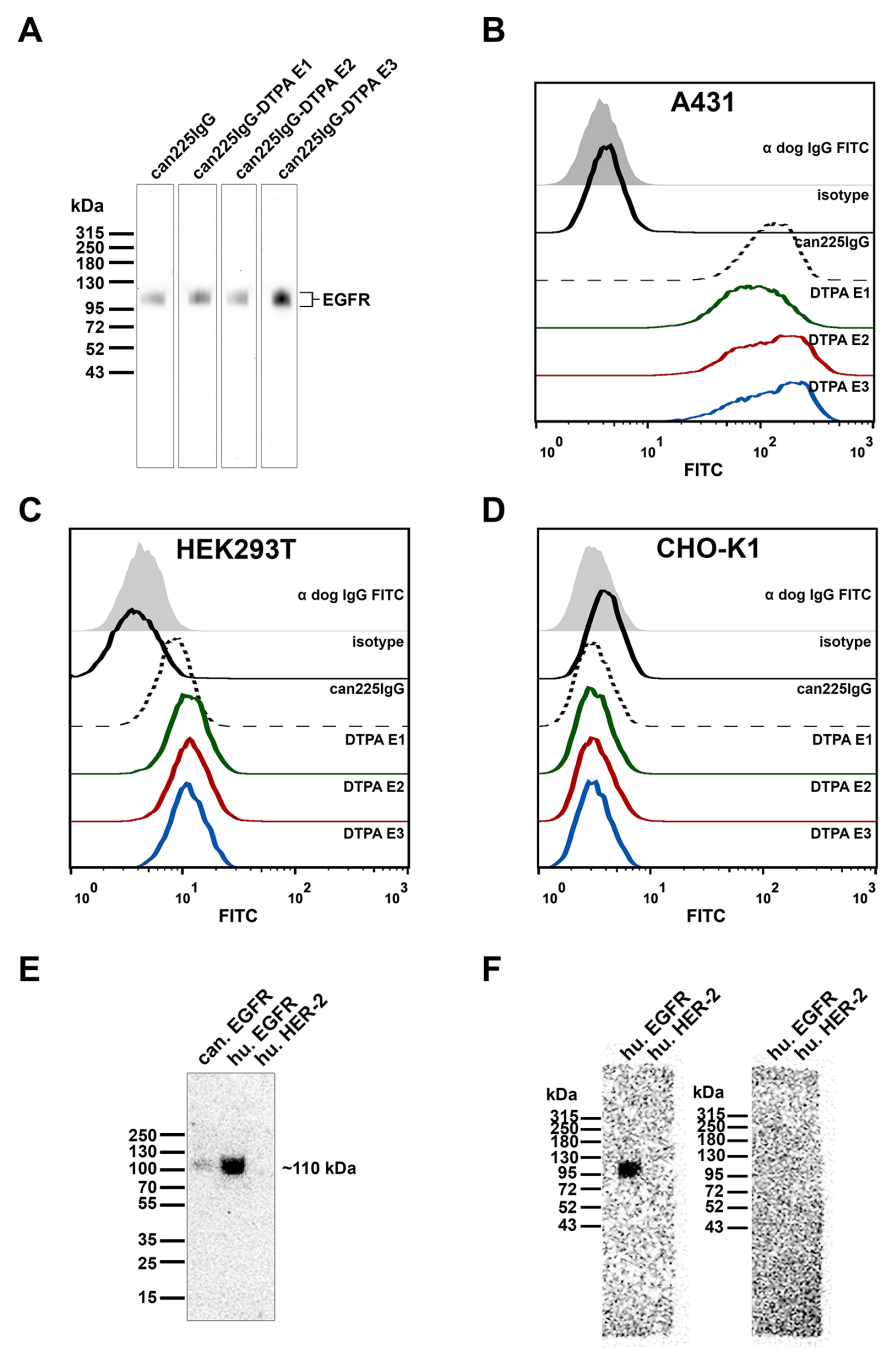
Specificity of DTPA-conjugated can225IgG variants E1-E3 **(A)** Western blot with soluble recombinant human EGFR as sample, detected with unmodified (lane 1) or DTPA-conjugated can225IgG variants E1-E3 (lanes 2-4), followed by anti-dog IgG HRP. **(B)** Flow cytometric binding analysis of unmodified can225IgG versus DTPA-conjugated can225IgG variants on EGFR-overexpressing A431 cells, detected by FITC-conjugated anti-dog IgG. **(C)** Flow cytometric binding analysis of unmodified can225IgG versus DTPA-conjugated can225IgG variants on moderately EGFR-expressing HEK293T cells. **(D)** Flow cytometric binding analysis of unmodified can225IgG versus DTPA-conjugated can225IgG variants on EGFR negative CHO-K1 cells. **(E)** Radioblot with canine EGFR, human EGFR and human HER-2, detected with ^99m^Tc-DTPA-can225IgG. **(F)** Left panel: radioblot with human EGFR and human HER-2, detected with ^99m^Tc-DTPA-can225IgG, right panel: radioblot with human EGFR and human HER-2, pre-blocked with 10μg/ml unlabeled can225IgG and subsequently detected with ^99m^Tc-DTPA-can225IgG.

Since DTPA has a several times higher molecular mass than an amino acid, it seemed likely that functionalization in the variable region might negatively influence the specificity and affinity of the antibody towards EGFR. Therefore, it was essential to re-evaluate specificity upon DTPA-conjugation and radiolabeling. First, all 3 DTPA-conjugated variants were tested head to head with the original antibody on a preparative western blot. Here, all tested specimens retained their reactivity towards EGFR (Figure 2A).

Next, binding properties to native EGFR on the surface of cancer cells was investigated via flow cytometry. On endogenously high EGFR expressing A431 cells (Figure 2B), all 3 functionalized antibody probes bound to EGFR to the same extent as the positive control can225IgG (median fluorescence intensities: can225IgG 125; E2 128; E3 132), only sample E1 showed slightly less binding (median fluorescence intensity 85.3) than the others. However, this observation of slightly reduced staining could not be detected in EGFR^lo^ HEK293T cells. Here, all 3 functionalized antibody specimens stained with comparable fluorescence intensity (Figure 2C). As anticipated, none of the antibodies (original or modified) bound to the EGFR negative cell line CHO-K1 (Figure 2D). Since variant E2 appeared to be optimal in assays investigating specificity as well, we decided to perform all subsequent radiolabeling experiments with this compound.

^99m^Tc-labeling resulted in a mean radiochemical yield of 44.34% ±15.28 and a mean specific radioactivity of 3707 GBq/μmol (Table 2). Metal complexation may lead to a significant change in the pharmacological properties of an antibody; therefore we re-evaluated the specificity of ^99m^Tc-DTPA-can225IgG on a radioblot. ^99m^Tc-DTPA-can225IgG (E2) successfully detected recombinant soluble canine EGFR and recombinant soluble human EGFR, but did not bind to another member of the human epidermal growth factor receptor family, HER-2 (Figure 2E). Interestingly, the in-house produced canine EGFR detected by ^99m^Tc-DTPA-can225IgG showed a less intense band on the blot compared to the commercially available human EGFR. This effect was reproducible when repeating the experiment and could not be traced back to uneven loading. To affirm that the epitope specificity of the radiolabeled antibody stayed unchanged, we performed a blocking experiment by pre-incubation one of two identical membranes with cold (non-radioactive), unmodified can225IgG. ^99m^Tc-DTPA-can225IgG specifically detected EGFR on the non-blocked blot corresponding to the results in Figure 2E. The blocked blot, however, was lacking the signal at the EGFR band, further demonstrating that the radio-compound preserved its epitope-specificity (Figure 2F).

**Table 2:** Summary of radiolabeling reactions of ^99m^Tc-DTPA-can225IgG

	n	Unit	Mean	SD	Median
**Starting activity**	13	GBq	0.96	0.41	1.07
**Product activity**	13	GBq	0.35	0.19	0.42
**Yield (decay corrected to start)**	13	%	44.34	15.28	47.86
**Product concentration**	5	nM	59.59	19.79	56.23
**Total volume of product**	5	ml	2.96	1.13	3.00
**Specific activity**	5	GBq/μmol	3707	1778	2816

n – number of experiments analyzing the depicted parameter.

### Radio- and protein stability of ^99m^Tc-DTPA-can225IgG

The major aim of our study was to develop a diagnostic compound for potential *in vivo* use in canine cancer patients. Accordingly, we intended to identify the ideal formulation that guarantees sufficient stability for the interval between radiolabeling and intravenous application (bolus of 1-3 ml). Under optimal conditions, we expected an interval of 45-70 minutes from quality control-release of the radiolabeled antibody until administration; in fact, we tested stability of the antibody for up to 4 hours in order to gain a more detailed picture on its pharmacokinetic stability. We assessed the amount of intact radiolabeled antibody formulated in TRIS-buffered saline (TBS, pH 7.4), 0.9% NaCl, NaOAc (100 mM) pH 6.0 or pH 7.0, respectively.

**Figure 3:**
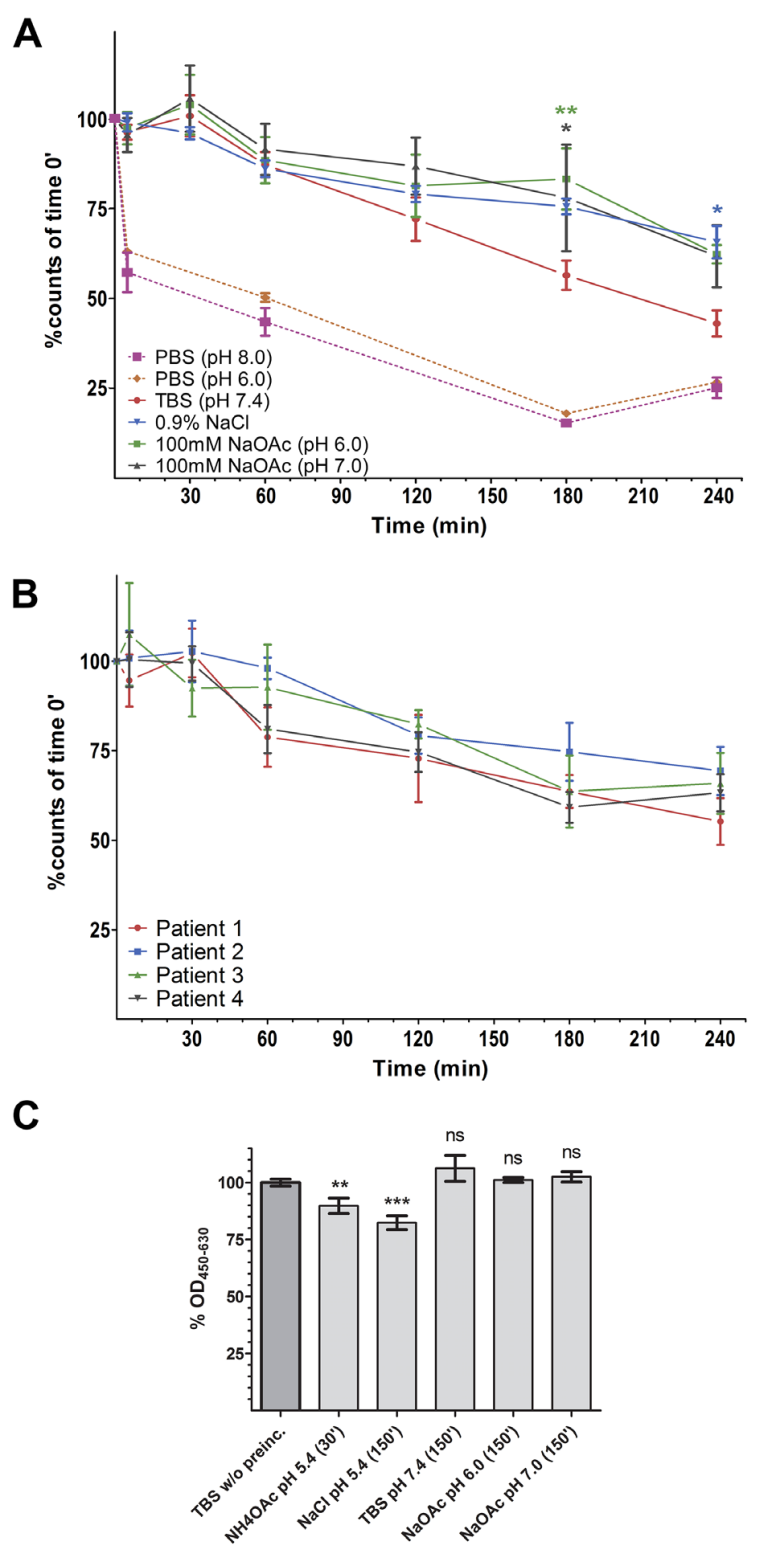
Radio- and protein-stability of ^99m^Tc-DTPA-can225IgG **(A)** % of intact ^99m^Tc-DTPA-can225IgG in various buffers, normalized to time point 0. Incubation was carried out at room temperature. Dotted pink line: PBS (pH 8.0) (n=2), Dotted orange line: PBS (pH 6.0) (n=2), Solid red line – TBS (pH 7.4), blue – 0.9% NaCl, green – NaOAc pH 6.0, grey – NaOAc pH 7.0. Denoted significances refer to the buffer indicated by the color, compared to TBS; n=3. **(B)** % of intact ^99m^Tc-DTPA-can225IgG in canine mammary carcinoma patient’s sera, samples were incubated at 37°C; n=4. **(C)** % Captured can225IgG from of pre-incubated buffer samples, normalized to freshly prepared can225IgG. Statistical significances refer to differences in bound can225IgG compared to the reference sample; n=3.

As pre-experiments had shown poor stability in phosphate buffered saline (PBS, pH 6.0 and 8.0) we discontinued stability testing with this buffer and did not include it in statistical tests (Figure 3A, dotted lines).

We could not observe any statistically significant difference between the other four tested formulations up to an incubation time of at least 3h (Figure 3A). At 60 minutes after radiolabeling, a likely time point for administration to the patient, still 87.11% of ^99m^Tc was coupled to the antibody incubated in TBS (Table 3).

**Table 3:** Stability of ^99m^Tc-DTPA-can225IgG in various buffer formulations

Time	TBS (pH 7.4)	0.9% NaCl	NaOAc (pH 6.0)	NaOAc (pH 7.0)
**60’**	87.11% ±6.04	85.97% ±4.01	88.35% ±11.08	91.38% ±12.24
**120’**	71.95% ±10.47	78.97% ±3.83	81.23% ±15.00	86.69% ±13.70

Amount of intact ^99m^Tc-DTPA-can225IgG in different buffer formulations 60 and 120 minutes after radiolabeling. Data depicted in %, normalized to time point 0.

In a second step, we employed a similar experimental setting in order to simulate compound stability in dog serum. We used undiluted sera of four canine mammary carcinoma patients (patients' characteristics in Supplementary Table 2) and monitored the amount of tracer at 37°C for up to 4 hours. Although we observed some patient-to-patient variations, no statistically significant differences were detectable within this time frame (Figure 3B). Notably, even after 4 hours, still all patient sera contained at least 55% of intact ^99m^Tc-DTPA-can225IgG.

We experienced a strongly reduced to completely diminished binding to EGFR when the radio-compound was formulated in 0.9% NaCl or NaOAc and stored in this formulation for several hours. Upon closer investigation, we found that these buffers were not capable of fully neutralizing the remaining NH_4_OAc buffer after the PD-10 purification, resulting in a lower pH of 5.4-5.7. We assumed that this low pH may account for partial protein denaturation which negatively influences antibody binding. Consequently, an enzyme-linked immunosorbent assay (ELISA) was performed: EGFR was coated as a capture antigen, followed by incubation with the unmodified original can225IgG being pre-incubated in the respective buffers, or freshly diluted can225IgG as normalization control.

The antibody was pre-incubated for 30’ in the NH_4_OAc buffer used during the ^99m^Tc-labeling in order to simulate the conditions during radiolabeling. All other pre-incubations were carried out for 2.5h, which is the expected maximum duration of all *in vitro* experiments of this study.

We observed approximately a 10% drop of binding in the NH_4_OAc sample and a 20% decrease in the 0.9% NaCl formulation (Figure 3C). TBS (pH 7.4) seemed to be superior regarding protein stability, though not significantly. Moreover, TBS never displayed insufficient neutralization following radiolabeling. Thus, we concluded that TBS is the most suitable formulation and performed all further experiments onwards using this buffer system.

### Autoradiography of dog mammary carcinoma tissue

**Figure 4:**
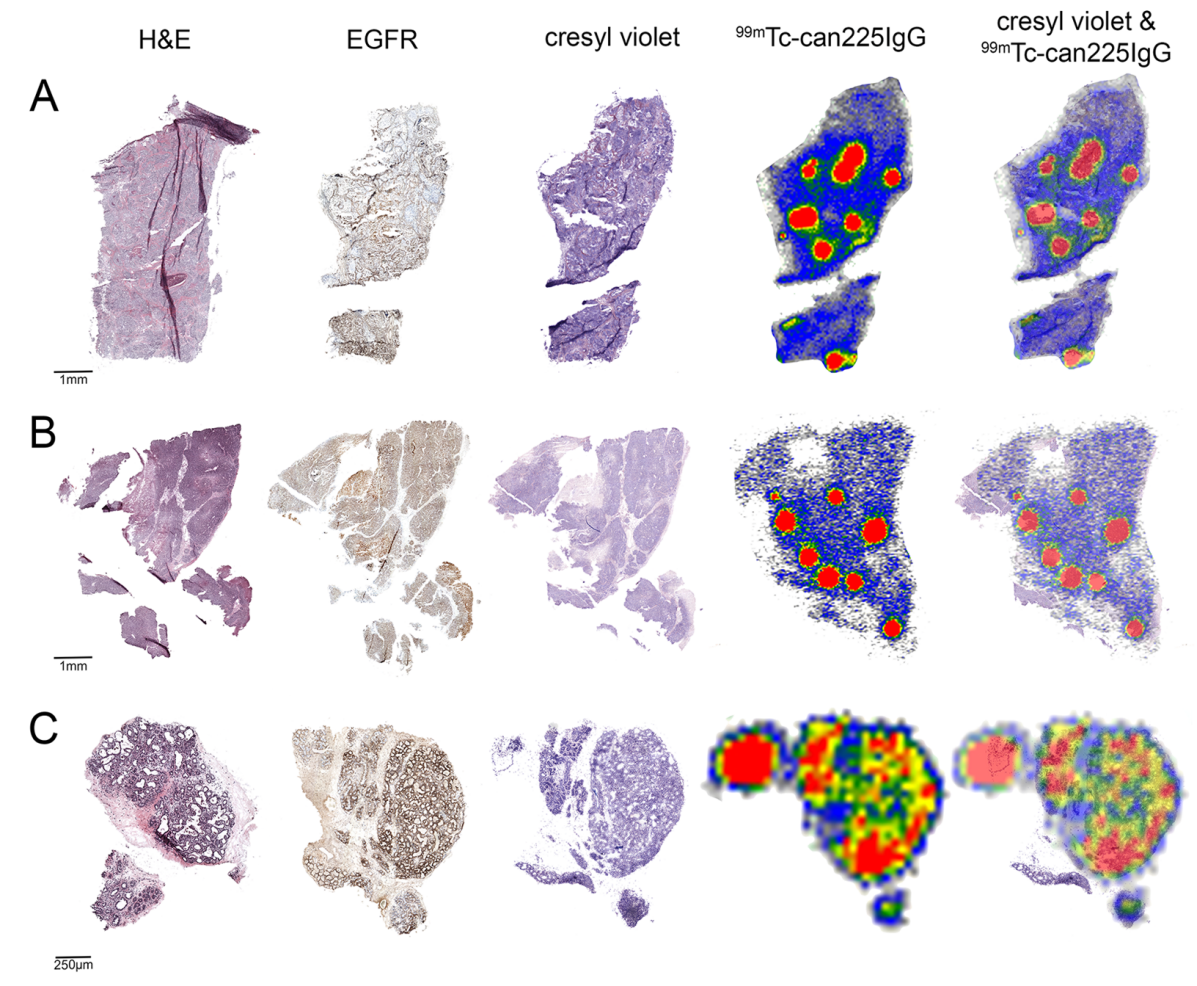
Immunohistochemistry and autoradiography of canine mammary carcinoma sections 5 μm tumor sections of 3 canine mammary carcinoma patients **(A-C)** were stained with hematoxylin/eosin (first column) and for EGFR expression (second column). 10 μm sections of the same tumor were used for autoradiography with ^99m^Tc-DTPA-can225IgG (fourth column). The same sections used for autoradiography were stained with cresyl violet in order to visualize tissue morphology (third column) and were used to generate an overlay (fifth column).

Autoradiography of canine mammary carcinoma tissue sections was performed in order to confirm binding of ^99m^Tc-DTPA-can225IgG to native canine EGFR in tissue. Immunohistological staining confirmed EGFR overexpression in all tested tissue samples in a membrane-specific manner (Figure 4, second column; Supplementary Figures 1 and 2). The autoradiography of a subsequent section showed moderate to high uptake in the tissue. Here, activity was mainly concentrated at regions being highly positive for EGFR expression. (Figure 4 fourth and fifth column). The signal in row C, column 4 and 5, appears blurry as it was recorded at the resolution limit of this technology. Patients' characteristics are disclosed in Supplementary Table 3.

### Real-time kinetics determination of ^99m^Tc-DTPA-can225IgG

**Figure 5:**
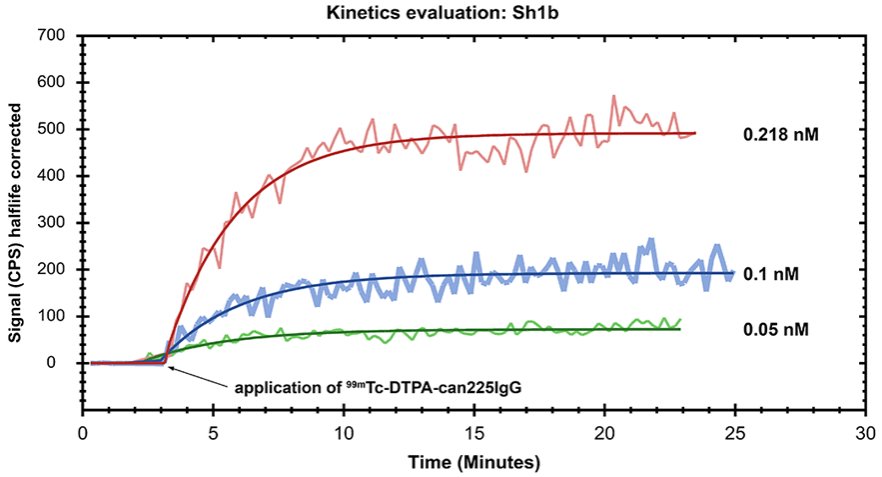
Affinity determination of ^99m^Tc-DTPA-can225IgG towards canine EGFR The EGFR+ canine mammary carcinoma cell line Sh1b was used to determine the affinity of ^99m^Tc-DTPA-can225IgG in a Ligand Tracer® approach. Real-time association was measured by incubation of the cells with 0.05 nM (green line), 0.1 nM (blue line) and 0.218 nM (red line) ^99m^Tc-DTPA-can225IgG until equilibrium was reached. Binding kinetics were calculated using a 1 to 1 ligand-target fitting algorithm.

In order to determine real-time kinetics and affinity of _99m_Tc-DTPA-can225IgG towards native canine EGFR, a competition binding assay using Ligand Tracer® Yellow was performed and the uptake of the radioactive compound in Sh1b canine mammary carcinoma cells was measured. Using 3 different concentrations of the compound, we could determine a K_D_ of 0.8 ±0.0031 nM towards naturally expressed canine EGFR (Figure 5). The respective dissociation rate constant k_d_ has been determined as 4.93e^-3^ ±1.61e^-5^ and the association rate constant k_a_ as 5.66e^6^ ±1.55e^3^.

## DISCUSSION

Currently, decisions for patient stratification in clinical oncology heavily depend on histopathology. Especially for specific treatment of tumors with targeted therapies such as monoclonal antibodies, companion diagnostics evolved, determining the expression of a tumor-associated antigen (TAA) in the malignant tissue. Immunohistochemical *in vitro* diagnostic tests are available on the market for detection of EGFR overexpression, classifying tissues from EGFR negative to +++ positive [18].

While histopathology is most often obtained from a single biopsy site, and PET scans localize tumors throughout the body via their metabolic activity, information on the different EGFR expression levels on primary or secondary lesions could provide important information to the clinical oncologist and have direct implications on targeting strategies.

For management of neuroendocrine tumor patients, this strategy is already a cornerstone in clinical decision-finding. As neuroendocrine tumors have relatively high expression levels of somatostatin-receptors (SSTRs) [19], several specific tracers were developed. The most commonly applied is the SSTR-ligand [^111^In-DTPA^0^]octreotide, which is used for diagnosis and staging. Tumors being positively marked by [^111^In-DTPA^0^]octreotide can be treated subsequently with SSTR-ligand bound therapeutic radionuclides, such as [^90^Y-DOTA0-Tyr^3^]octreotide (^90^Y-DOTATOC) and [^177^Lu-DOTA0-Tyr^3^]octreotate [20–22]. Such combinations of diagnostically- and therapeutically-labeled molecules are often called “theranostic twins” [23].

The aim of this study was to establish a similar diagnostic option in a comparative medicine setting using can225IgG, a caninized version of the widely applied monoclonal antibody cetuximab. We could demonstrate that upon functionalization with DTPA, can225IgG can be effectively radiolabeled with ^99m^Tc, a diagnostic SPECT-nuclide (Figures 1 and 2). Throughout all these modification steps, ^99m^Tc-DTPA-can225IgG retained its specificity towards EGFR in solid phase (Figure 2E-2F), on the surface of cancer cells in solution (Figure 2B-2D) as well as in canine mammary carcinoma tissue (Figure 4). Furthermore, we could determine the affinity of ^99m^Tc-DTPA-can225IgG in real-time competition binding assays using the LigandTracer® Yellow. The obtained K_D_ value of 0.8 nM corresponds to a high-affinity binding typical of therapeutic monoclonal antibodies. Cetuximab, the human-mouse chimeric antibody that served as a template for can225IgG, has a K_D_ value of 0.147 nM towards EGFR expressed on A431 cells and 0.2 nM determined using recombinant soluble EGFR [14]. Considering that the cetuximab binding epitope of canine EGFR differs in 4 amino acids [13] and the possible modifications through the functionalization procedure, the K_D_ value we observed for ^99m^Tc-DTPA-can225IgG is astonishingly good. The high affinity of ^99m^Tc-DTPA-can225IgG to canine EGFR confirmed that the lower signal intensity observed in blots (Figure 2E) is most probably not a result of lower affinity of the antibody, but rather accountable to the difficulties encountered during the production of canine EGFR and its low stability. Nevertheless, these results do not provide any information about the *in vivo* kinetics of ^99m^Tc-DTPA-can225IgG; the rate of tissue penetration and the uptake in tumors will have to be experimentally determined in the course of a clinical trial in canine carcinoma patients.

Considering direct anti-tumor effects of the antibody via growth signal inhibition or immune cell activation, we do not expect direct targeting effects due to the extremely low *in vivo* concentration of this tracer immunoglobulin. Typically, the dosages of radioactive antibody tracers (for diagnostic purposes) range between 0.1-0.4 GBq [24], which would be approximately 10-30 μg can225IgG, considering the specific activity we achieved in this study. For an average canine patient of 20 kg (0.744 m^2^ body surface area [25]) as an example, the dosage roughly equals 0.04 mg/m^2^. On the contrary, the approved therapeutic dose of cetuximab for human patients is 400 mg/m^2^ as an initial dose, followed by 250 mg/m^2^ as weekly maintenance dose [26]. Presumably, using an up to 10.000-fold lower antibody concentration than the current clinically applied doses would not show any signal inhibition or immunological effect in an *in vivo* model or in a clinical study with canine patients.

We could demonstrate that ^99m^Tc-DTPA-can225IgG is stable in four different buffers (Figure 3A), all of which are suitable for intravenous injection. As radiolabeling facilities and SPECT-cameras can be in different locations, it was crucial to prove the stability of ^99m^Tc-DTPA-can225IgG for transportation and short term storage at room temperature. Our results indicate that under neutral pH the reagent can be stored for up to 1h, which should be suitable for transportation as well as patient preparation.

Another important point was to determine whether ^99m^Tc-DTPA-can225IgG would be also stable in serum following intravenous administration. Here we could demonstrate stability with non-significant patient-to-patient variability for up to four hours, although these serum samples were derived from four different dog breeds.

Furthermore, after 4 hours, more than 55% of ^99m^Tc-DTPA-can225IgG was still intact in all tested serum samples. A study by Ping et al. [27] demonstrated in a mouse model, that 20h after administration uptake of ^64^Cu-DOTA-cetuximab in EGFR-positive tumor tissue was visible in PET. Although the *in vitro* stability of ^99m^Tc-DTPA-can225IgG suggests trans-chelation to serum proteins, a positive performance for imaging is not discarded yet. A previous report [28] evidenced successful PET imaging of HER2 in a mouse model of ovarian carcinoma, despite the poor stability in serum of the ^89^Zr-labeled antibody utilized. We thus assume that ^99m^Tc-DTPA-can225IgG may be stable enough for *in vivo* imaging; however the optimal time point to scan the patient has still to be determined in clinical trials. Therefore, we propose ^99m^Tc-DTPA-can225IgG a valuable tool to introduce TAA-specific whole body imaging in veterinary oncology.

Based on the outcome of these future scans, one would not only be able to precisely localize EGFR+ tumors in the individual patients, but also collect valuable information on the pharmacokinetics of tumor-targeting monoclonal canine IgGs for the first time. This could further advance the development of specific EGFR-targeting antibodies, such as can225IgG, for dog tumor patients. As this antibody could also be labeled with therapeutic radionuclides, e.g. Yttrium-90 (^90^Y) or Lutetium-177 (^177^Lu) “theranostic twins” could also be developed leading to highly specific as well as effective anti-cancer therapies.

Simultaneously, these clinical trials with dog patients suffering from spontaneously occurring tumors would provide insights for a possible translation of this approach back to human oncology, or to other species, like cats, that are also heavily affected by EGFR-overexpressing malignancies [29]. Also here, this novel theranostic approach could exploit imaging options in order to select patients more effectively.

## MATERIALS AND METHODS

### Cell lines, can225IgG and antigens

The human EGFR-overexpressing epithelial carcinoma cell line A431 (ATCC® CRL-1555™), the human embryonic kidney cell line 293T (ATCC® CRL3216™) and the chinese hamster ovary cell line CHO-K1 (ATCC® CCL-61™) were obtained from the American Type Culture Collection (ATCC, Manassas, VA, USA). The canine mammary carcinoma cell line Sh1b was kindly provided by Prof. Dr. Gerard Rutteman from the Department of Clinic Science and Companion Animals, University of Utrecht, Utrecht, the Netherlands. A431 and HEK293T were cultured in Dulbecco`s Modified Eagle Medium (cat# 11995065, Gibco), supplemented with 10% fetal bovine serum (cat# 10270106, Gibco), 2 mM Glutamine (cat# 25030081, Gibco), 100 U/ml Penicillin and 100 µg/ml Streptomycin (cat# 15140122, Gibco); CHO-K1 was grown in Ham’s F-12 Nutrient Mix (cat# 21765029, Gibco) supplemented with 10% fetal bovine serum (cat# 10270106, Gibco), 100 U/ml Penicillin and 100 μg/ml Streptomycin (cat# 15140122, Gibco) and Sh1b was cultured in Ham’s F-12 Nutrient Mix (cat# 21765029, Gibco) supplemented with 10% fetal bovine serum (cat# 10270106, Gibco) and 10 μg/ml gentamycin sulphate (cat# 2475.1, Carl Roth). The cells were grown at passages 5-17 at 37°C under a humidified atmosphere with 5% CO_2_. 293T has been freshly purchased from ATCC and the identity of A431 has been authenticated by Multiplexion (Heidelberg, Germany) prior to experiments.

Can225IgG was produced in CHO DUKX-B11 cells as described by Singer, Fazekas et al. [16].

Recombinant soluble human EGFR was purchased from ACROBiosystems (cat# EGR-H5222).

Recombinant soluble canine EGFR was produced in HEK293T cells. Detailed information about the expression and the sequence is disclosed in the Supplementary Materials and Supplementary Table 1.

Recombinant soluble human HER-2 was produced by Lec-1 cells [30], which were kindly provided by Prof. Daniel Leahy from Johns Hopkins University School of Medicine (Baltimore, Maryland, USA).

### Structural modeling of can225IgG

Modeling of can225IgG was done by using MODELLER version9v8, as described in Singer, Fazekas et al. [16]. p-SCN-Bn-DTPA was modeled by ChemDraw release 16.0 (Perkin Elmer, Waltham, MA, USA).

### Functionalization of can225IgG with DTPA

Can225IgG was functionalized for subsequent radiolabeling with S-2-(4-isothiocyanatobenzyl)-diethylene-triamine-pentaacetic acid (p-SCN-Bn-DTPA, in following ‘DTPA’) at piCHEM (Graz, Austria). For this reaction, DTPA was dissolved in DMSO at a concentration of 10 mg/ml. Can225IgG (2 mg/ml, dissolved in 0.1 M PBS, pH 8.0) was mixed with DTPA at a molar excess defined below (E1-3). The reaction mix was then allowed to react at room temperature for 2 h and then gently shaken at 4°C over night. The next day, the reaction mix was diluted with PBS pH 7.2 and excess of the chelator was removed by G25 Sephadex size exclusion purification (PD-10 column, cat# 17-0851-01, GE Healthcare). Fractions containing the DTPA labeled antibody were eluted with 0.1 M PBS pH 7.2 and collected. In order to optimize the functionalization of the antibody for subsequent experiments, we tested 3 different experimental conditions (can225IgG E1-E3): can225IgG E1 was generated by using a 25:1 molar excess of DTPA to can225IgG; can225IgG E2 was functionalized with 50x molar excess of DTPA and finally, can225IgG E3 was also functionalized with a 50:1 molar ratio of DTPA to antibody, however, the reaction was carried out at a pH of 9.0 instead of 8.0.

### Radiolabeling of the DTPA-functionalized can225IgG with ^99m^Tc

^99m^TcO_4_^-^ was eluted from a ^99^Mo/^99m^Tc generator with sterile 0.9% NaCl. 200 μL of the eluate (typically with an activity of ∼1 GBq) was added to the reaction vessel, containing 400 μl 100 mM NH_4_OAc (pH 5.4), 50 μg DTPA-can225IgG and 3 μL of a reduction solution (2.4 mM SnCl_2_ (cat# 818150, Merck) and 4.25 mM L-ascorbic acid (cat# A5960, Sigma-Aldrich) in sterile 0.9% NaCl previously purged with argon). The reaction mixture was stirred for 25 min at room temperature (RT). The crude product was then purified with a Sephadex™ G25M PD-10 column (cat# 17-0851-01, GE Healthcare) and fraction-wise eluted with TBS (pH 7.4). Purity of the radiolabeled antibody was assessed via instant thin layer chromatography, using glass microfiber chromatography paper impregnated with silica-gel (iTLC-SG) (cat# SGI0001, Agilent Technologies, Santa Clara, CA, USA) and 0.9% NaCl as mobile phase. In order to obtain the precise specific activity for each radiolabeling batch, protein concentration of the final product was determined according to the microassay protocol of the Quick Start™ Bradford Protein Assay (cat# 5000201, Bio-Rad Laboratories) using purified canine IgG (cat# IR-DG-GF, Dunn Labortechnik) for the standard curve.

### PAGE and immunoblots

All gels and blots were performed using 4–15% Mini-PROTEAN® TGX™ precast gels (cat# 4561084, Bio-Rad Laboratories), at running conditions of 200 V for 32 minutes in standard PAGE buffer by Laemmli. Molecular weight markers used: PageRuler Plus prestained protein ladder, 10-250 kDa (cat# 26612, ThermoFisher) and Spectra™ Multicolor high range protein ladder, 40-300 kDa (cat# 26625, ThermoFisher, lot# 00316364). Gels for the molecular weight analysis were stained with SimplyBlue™ SafeStain (cat# LC6060, Life Technologies) according to the manufacturer’s instructions.

For western and radio-blots, proteins were blotted onto 0.2 μm pore-sized nitrocellulose membranes (cat# 10600001, GE Healthcare) using a semi-dry blotting device (Bio-Rad Laboratories) at a current of 80 mA/gel for 60 minutes. Subsequently, membranes were blocked by 5% skimmed milk powder solubilized in TBST (50 mM TRIS, 150 mM NaCl, 0.1% Tween-20, pH 7.4) for 60 min at RT.

To verify antibody integrity, unmodified and DTPA-conjugated can225IgG was detected by HRP-labeled anti-dog IgG(Fc) antibody (cat# 304-035-008, Jackson ImmunoResearch Europe), diluted 1:5000 in blocking buffer and incubated for 60 minutes at RT. Following washing with TBST (3×10 minutes), membranes were developed with an ECL substrate (cat # RPN2232, GE Healthcare) using a CCD-based VersaDoc Imaging System (Bio-Rad Laboratories, Hercules, CA, USA).

Soluble human EGFR was detected stripe-wise by unmodified can225IgG and by can225IgG-DTPA E1-E3 at concentrations of 1 μg/ml in blocking buffer and incubated for 60 min at RT. Upon washing with TBST, the membrane was incubated with HRP-labeled anti-dog IgG(Fc) antibody (1:5000, diluted in blocking buffer) for 60 min at RT. After a final washing step, the membrane was developed as described above.

Radioblots were incubated with approximately 100-200 kBq ^99m^Tc-DTPA-can225IgG diluted in blocking buffer for 1h at RT. The pre-blocked radioblot (see Supp. Figure 3 for detailed assay scheme) was additionally pre-incubated with 10 μg/ml unlabeled can225IgG prior to incubation with the radiolabeled antibody. Following incubation with the radiolabeled compound, blots were washed 3 times 5’ with TBST and imaged using an Instant Imager v1.27 (Packard Instruments Company, Meriden, CT, USA).

### Mass spectroscopy

Mass spectroscopy analysis was carried out on a Bruker Microflex MALDI-TOF device using the software ‘flexControl’ v2.4 (Bruker, Billerica, MA, USA). Antibody solutions (∼0.2 μg/ml) were dotted on a sinapinic acid matrix (cat# 8201345, Bruker) and ionized at 81% laser power. Mass spectra were converted to mzML using MSConvertGUI (ProteoWizard) and analyzed using mMass v5.5.0 (© Martin Strohalm). Baseline correction parameters: Precision 15, Relative offset 25. Smoothing window size: 40% of main peak width at 50% intensity, can225IgG light chain: 32, heavy chain: 404, can225IgG-DTPA light chain: 350, heavy chain: 637.

### Flow cytometry

3x10^5^ cells were stained with 10 µg/ml μg of either unmodified can225IgG or one variant of the functionalized can225IgG E1-E3 or isotype antibody (canine IgG standard, cat# IR-DG-GF, Dunn Labortechnik) in PBS+2% normal goat serum (NGS) for 30 min on ice. Following a washing step with PBS+2% NGS, bound dog IgG was detected with 10 µg/ml FITC-labeled anti dog IgG (cat# 304-095-008, Jackson Immuno Research), incubated for 30 min on ice and followed by 2 washing steps with PBS+2% NGS. Subsequently, 10.000 single/viable cells were recorded on a FACSCalibur™ (BD Biosciences, Franklin Lakes, NJ, USA). Data analysis was performed using FlowJo v10.0.7 software (Flow Jo LLC, Ashland, OR, USA).

### Serum/buffer stability; instant thin-layer chromatography

In order to determine the amount of intact tracer over time and to find an ideal formulation for a small intravenous bolus application, ^99m^Tc-DTPA-can225IgG was incubated in various buffers and in dog serum. Sera were obtained in the course of diagnostic workups at the Oncology Unit at the Small Animal Hospital, University of Veterinary Medicine Vienna (Vienna, Austria). Buffer mixtures were then incubated at room temperature and sera at 37°C in order to simulate storage and transport conditions or *in vivo* stability. Stability was monitored for 4 hours altogether. At specific time points, 2 μl aliquots of the incubation mixtures were analyzed by instant thin layer radio-chromatography using 0.9% NaCl as mobile phase. Subsequently, dry chromatography sheets were read out using the Instant Imager v 1.27 (Packard Instruments Company, Meriden, CT, USA) and processed with ‘Imager’ software, version 2.05 for Windows 95 (Packard Instruments Company, Meriden, CT, USA). The amount of intact tracer was determined as the percentage of total radioactivity bound to the silica gel paper (R_f_=0), whereas the amount of ^99m^TcO_4_^-^ was determined using the radioactivity moved with the solvent front (R_f_=1). Stability of the compound was normalized to the amount of intact tracer measured at time point 0. All stability tests were performed in quadruplicates.

### Enzyme-linked immunosorbent assay (ELISA)

96-well Maxisorp Nunc-immunoplates (cat# 442404, ThermoFisher) were coated over night with 1 μg/ml human soluble EGFR. Following blocking with the TBS-based SuperBlock blocking buffer (cat# 37535, ThermoFisher), 1 μg/ml solutions of can225IgG were applied to the plate in triplicates (samples: freshly diluted can225IgG in TBS, 30 min pre-incubated can225IgG in NH4OAc, 150 min pre-incubated can225IgG in 0.9% NaCl (pH 5.4), TBS (pH 7.4), 100 mM NaOAc (pH 6.0) and 100 mM NaOAc (pH 7.0)). Following 1h incubation, bound can225IgG was detected by a HRP-labeled anti-dog IgG(Fc) antibody (cat# 304-035-008, Jackson ImmunoResearch Europe), 1:5000 diluted in TBS +0.05% Tween-20 +2% BSA. Bound detection antibody was visualized by TMB OptEia substrate (cat# 555214, BD Biosciences) and recorded at 450 nm (and 630 nm reference wavelength) using the Infinite® M200 microplate reader (Tecan, Männedorf, Switzerland).

### Immunohistochemistry

Canine mammary carcinoma tissue samples were purchased from the VetBioBank of the VetCore, Veterinary University of Vienna. For the immunohistochemical and H&E stainings, 5 μm thick sections of frozen canine mammary carcinoma samples were fixed with ice cold acetone for 10 minutes, then after air drying, washed in PBS for another 10 minutes. H&E staining was performed according to standard protocols. Slides for EGFR were fixed in 10% neutral buffered formaldehyde for 10 min and then stained with the DAKO EGFR PharmDX™ kit (Agilent Technologies) including isotype controls, according to the manufacturer’s instructions. Stained slides were subsequently mounted with Fluoromount (cat# F4680, Sigma-Aldrich) and recorded with the Tissue FAXS automated scanning system (TissueGnostics, Vienna, Austria) at 20x magnification.

### Autoradiography

10 μm thick, untreated frozen canine mammary carcinoma sections were thawn for 5 minutes at RT and then blocked in TBS+2% bovine serum albumin (BSA) for 30 min. The slides were then incubated with ∼30 kBq/section ^99m^Tc-DTPA-can225IgG for 1h at RT. Subsequently, slides were incubated 2x for 5 min in ice-cold TBS+2% BSA and then dipped 10x into ice-cold H2O followed by drying under a cold air stream. Dry slides were placed on multisensitive phosphor screens (cat# 7001724, Perkin Elmer) for 24h and then recorded in a Cyclone Plus phosphor imager and analyzed using the OptiQuant® software (Perkin Elmer).

### Cresyl violet staining

The same slides used for autoradiography (5-6 half times post staining) were fixed in PBS+10% formaldehyde for 10 min and then rinsed briefly in H_2_O. Slides were then stained for 30 min in a bath of cresyl violet staining solution (1% w/v cresyl violet (cat# C504, Sigma-Aldrich) and 1% glacial acetic acid in H_2_O) at 60°C. Following a brief rinse in H_2_O, slides were differentiated in subsequent baths of 70% EtOH and 95% EtOH followed by a quick dip into 100% EtOH. Slides were then cleared by 2 subsequent washes with xylene and mounted with Histofluid (cat# 6900002, Marienfeld Superior). All slides were then recorded with the Tissue FAXS automated scanning system at 20x magnification.

### Affinity determination using Ligand Tracer® Yellow

The canine mammary carcinoma cell line Sh1b was seeded at 1x10^6^ cells/ml (2.5 ml total volume) in 100 mm*20 mm tissue culture dishes (cat# 664160, Greiner Bio-One) and incubated overnight in a tilted position in order to facilitate cell growth at one side of the dish only. The next day, cell culture medium was changed and the dish was moved to horizontal position for 24 hours for the purpose of blocking free binding sites on the plastic dish by the fetal bovine serum found in the media. For real-time kinetics assessment, the Ligand Tracer® Yellow (Ridgeview Instruments, Uppsala, Sweden) was employed: after background measurement, we applied 2.5 ml DMEM with 0.05 nM, 0.1 nM or 0.218 nM ^99m^Tc-DTPA-can225IgG and measured radioactivity at the cell-covered and the cell-free pole of the dish for 3 seconds, with 2 seconds waiting time in between. The background-corrected signal was then employed to calculate the kinetics using the TraceDrawer software v 1.7.1 (Ridgeview Instruments, Uppsala, Sweden) using the 1:1 ligand-target model.

### Statistical testing

All statistical analyses were carried out using GraphPad Prism v5.00 for Windows. We employed two-way ANOVA with Bonferroni’s post-test (CI: 95%) to determine statistical significances in the stability-testing (Figure 3A-3B) and one-way ANOVA combined with Bonferroni’s post-test (CI: 95%) in order to compare experimental settings in regard of protein stability (Figure 3C). Statistical significance is defined as * (p<0.05); ** (p<0.01) and *** (p<0.001).

## SUPPLEMENTARY MATERIALS FIGURES AND TABLES



## Abbreviations

ADCC: antibody-dependent cellular cytotoxicity
ADCP: antibody-dependent cellular phagocytosis
[^18^F]FDG: 2-deoxy-2-[^18^F]fluoro-D-glucose
^99m^Tc: technetium-99m
BSA: bovine serum albumin
CT: computed tomography
DTPA: diethylene-triamine-pentaacetic acid
E1-3: experimental setting 1-3 (of functionalization)
EGFR: epidermal growth factor receptor-1
ELISA: enzyme-linked immunosorbent assay
HER-2: human epidermal growth factor receptor 2
ITLC: instant thin layer chromatography
k_a_: association rate constant
K_D_: equilibrium dissociation constant
k_d_: dissociation rate constant
MALDI-TOF: matrix-assisted laser desorption ionization – time of flight
NGS: normal goat serum
PAGE: polyacrylamide gel electrophoresis
PET: positron emission tomography
SPECT: single-photon emission computed tomography
SSTR: somatostatin-receptor
TAA: tumor-associated antigen
