# Supplementary material for: Development of a radiolabeled caninized anti-EGFR antibody for comparative oncology trials
Source: Oncotarget. 2017 Sep 15;8(47):83128–41. doi: 10.18632/oncotarget.20914 (PMC5669955; doi:10.18632/oncotarget.20914)
Supplement: Supplementary file 1 [file oncotarget-08-83128-s001.pdf]

Development of a radiolabeled caninized anti-EGFR antibody for comparative oncology trials

SUPPLEMENTARY MATERIALS

Expression of recombinant canine EGFR

HEK293T cells were obtained from the American Type Culture Collection (293T (ATCC® CRL3216™)) and cultured in Dulbecco`s Modified Eagle Medium (cat# 11995065, Gibco), supplemented with 10% fetal bovine serum (cat# 10270106, Gibco), 2 mM Glutamine (cat# 25030081, Gibco), 100 U/ml penicillin and 100 µg/ml Streptomycin (cat# 15140122, Gibco) under humidified conditions (37°C, 5% CO_2). The codon optimized coding sequence of recombinant soluble canine EGFR (NCBI ref. seq. XP_533073.3, amino acids 1-618) was cloned into the pBudCE4.1 vector (cat# V53220, Invitrogen) under the EF-1α promotor using the NotI and KpnI restriction sites, including a 6xHIS tag. The complete DNA sequence is disclosed in Supplementary Table 1. Selection pressure was maintained using 100 µg/ml Zeocin™ (cat# R25001, Gibco). Clones were screened for productivity in a sandwich ELISA using cetuximab (Erbitux®, Merck) as capture antibody at 1 µg/ml, and a horseradish-peroxidase labeled Penta-HIS antibody (cat# 34460, Qiagen) at a 2000-fold dilution as a detection antibody. The best producing clone was then expanded in T-flasks and supernatant was collected in 5 day intervals. Binding/washing buffer (50 mM NaH_2PO_4, 500 mM NaCl, 0,05% Tween-20, pH 8.0) was mixed with the supernatant in a 1:2 ratio and subsequently loaded on a Ni sepharose HisTrap excel column (cat# 29-0485-86, GE Healthcare) at a constant flow rate of 1 ml/min. The column was then washed beads were washed with 20 column volumes of washing buffer (50 mM NaH_2PO_4, 500 mM NaCl, 10 mM imidazole, 0,05% Tween-20, pH 8.0). Bound canine EGFR was eluted with a constantly increasing concentration of elution buffer (50 mM NaH2PO4, 500 mM NaCl, 500 mM imidazole, 0,05% Tween-20, pH 8.0) and collected in 1ml fractions. Fractions containing canine EGFR were enriched using 30 kDa cut-off Amicon ultrafiltration tubes (cat# UFC903008, Millipore) combined with a buffer exchange against PBS, pH 7.4.

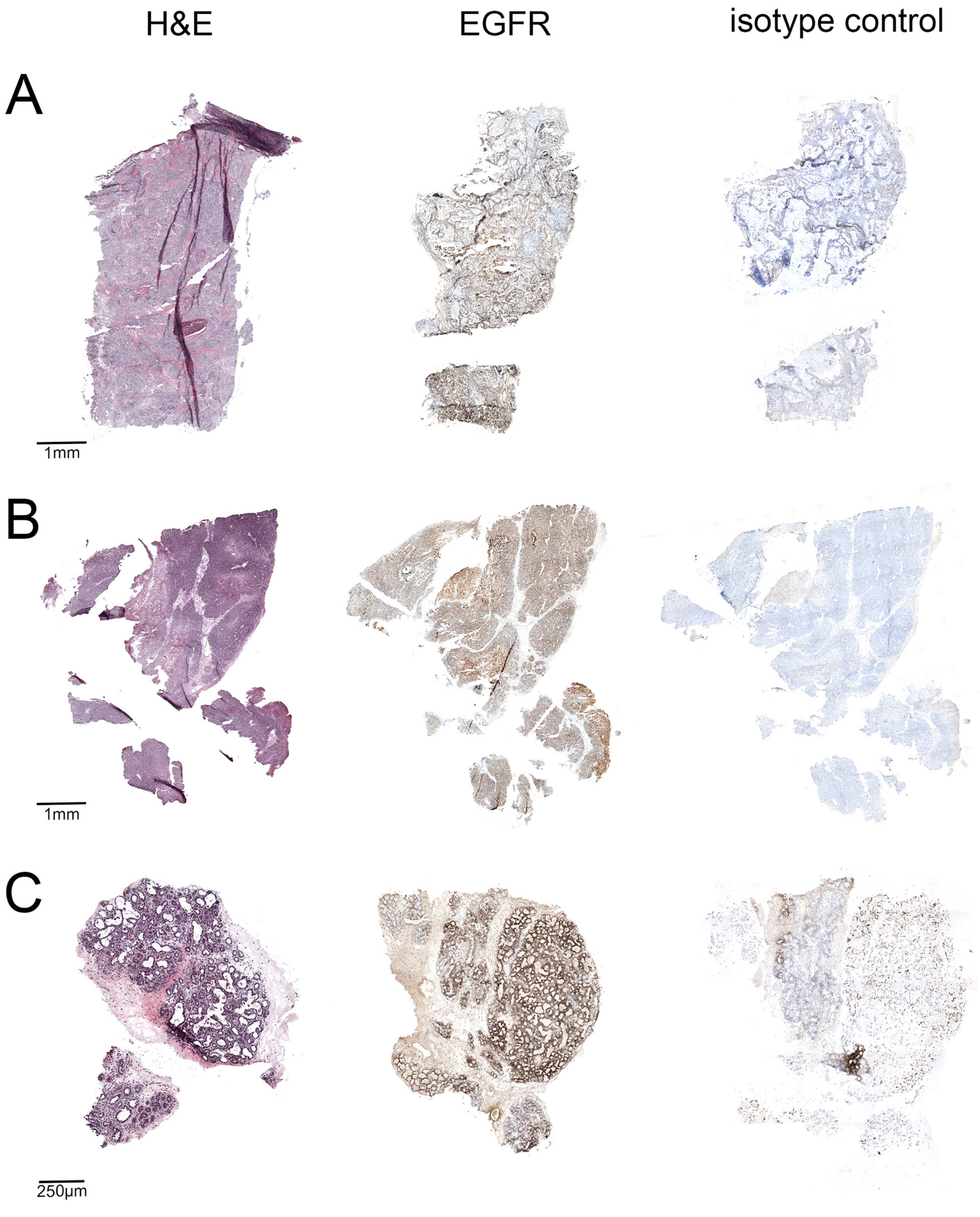

Supplementary Figure 1: EGFR+ canine mammary carcinoma sections with isotype control. Immunohistochemical staining of the tissue sections shown in Figure 4 of the main manuscript, including the isotype controls corresponding to the slides with EGFR staining. **(A-C)** refer to sections from the same patients as shown in Figure 3, patients' characteristics can be found in Supplementary Table 2.

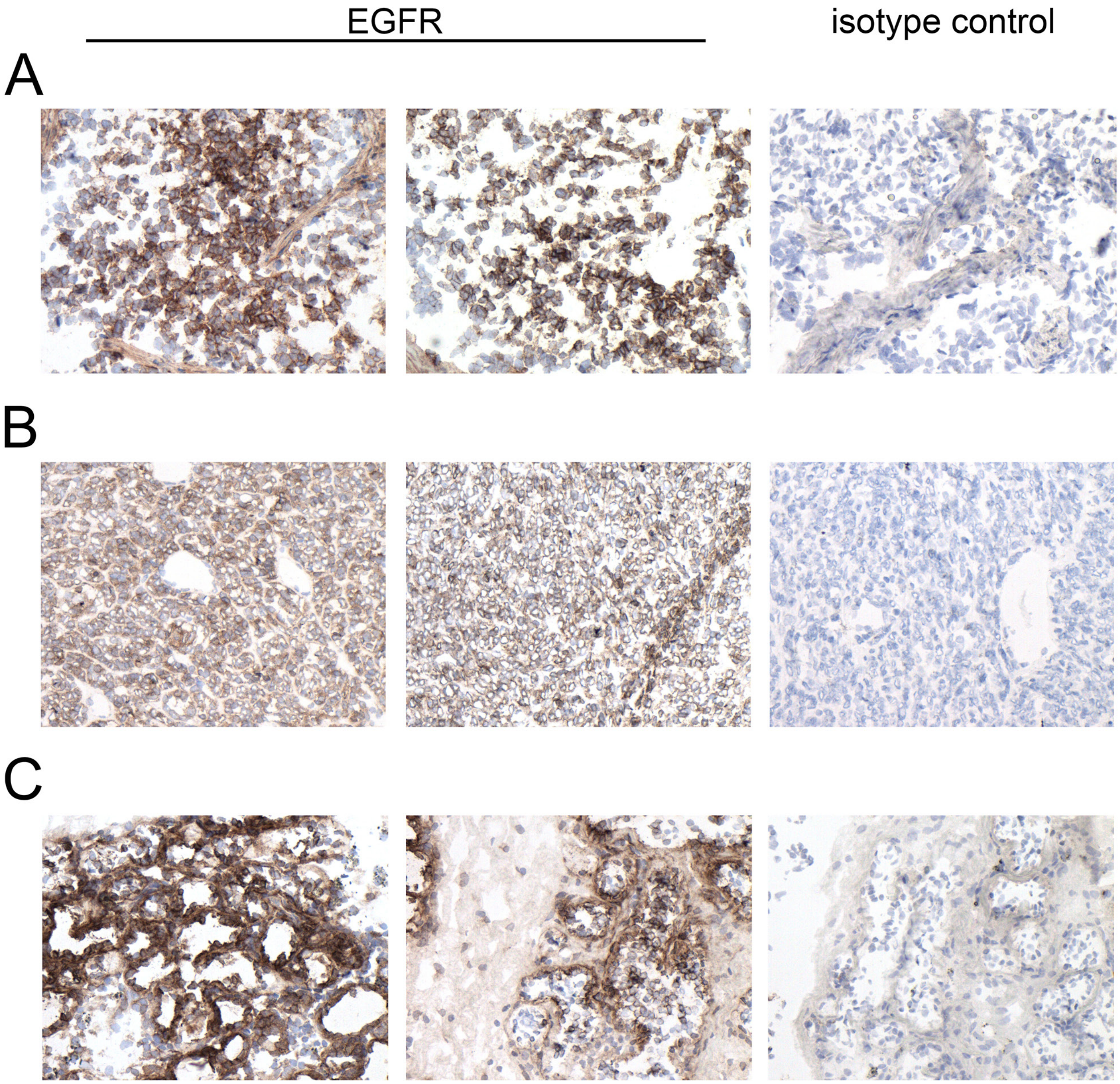

Supplementary Figure 2: EGFR+ canine mammary carcinoma sections, 20x magnification. Immunohistochemical staining of the tissue sections shown in Figure 4 of the main manuscript, 20x magnification. **(A-C)** refer to sections from the same patients as shown in Figure 3, patients' characteristics can be found in Supplementary Table 2.

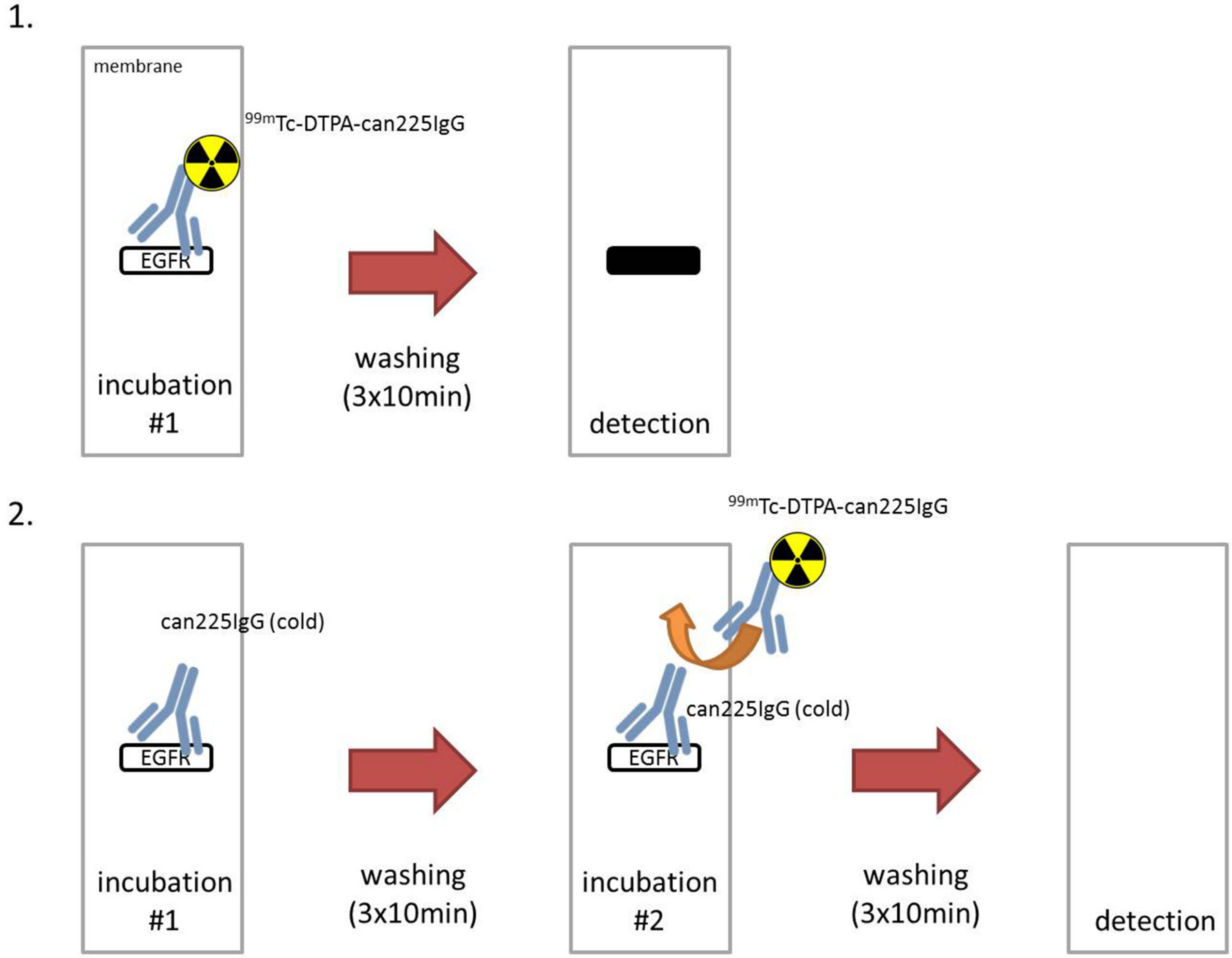

Supplementary Figure 3: Schematic diagram of the radioblot blocking assay shown in Figure 2E. In Figure 2E two identical blot membranes were loaded with EGFR, as depicted in row 1 and 2. 1.) The first membrane was incubated with ^{99m}Tc-DTPA-can225IgG, which bound to immobilized EGFR on the membrane, revealing a signal on the membrane. 2.) The second membrane was first incubated with cold (non-radioactive and non-functionalized) can225IgG at a high concentration (10µg/ml), which bound to immobilized EGFR and saturated all available epitopes. Following a washing step to remove unbound can225IgG, the membrane was again incubated, this time with ^{99m}Tc-DTPA-can225IgG. If no signal is obtained upon incubation with ^{99m}Tc-DTPA-can225IgG, its non-specific binding can be excluded.

Supplementary Table 1: Coding sequence of soluble canine EGFR

Feature	Sequence
**Human EGFR le<br>ader**<br>**Soluble canine EGFR (aa 1-618)**<br>**6xHIS tag STOP**	**ATGAGGCCTTCTGGAACAGCCGGCGCTGCTCTGCTGGCACTGCTGGCTGCTCTGT**<br>**GCCCTGCCTCTAGAGCC**ATGGCCGTGTGTCAGGGCACCAGCAACAGACTGACACA<br>GCTGGGAACCTTCGAGGACCACTTCCTGAGCCTGCAGCGGATGTTCAACAACTGC<br>GAGGTGGTGCTGGGCAACCTGGAAATCACCTACATGCAGCGGAACTACGACCTGA<br>GCTTCCTGAAAACCATCCAGGAAGTGGCCGGCTACGTGCTGATCGCCCTGAACAC<br>CGTGGAAAAGATCCCCCTGGAAAACCTGCAGATCATCCGGGGCAACGTGCTGTAC<br>GAGAACACCCACGCCCTGAGCGTGCTGAGCAACTACGGCAGCAACAAGACCGGCC<br>TGCAGGAACTGCCCCTGCGGAACCTGCACGAGATTCTGCAGGGCGCCGTGCGGTT<br>CAGCAACAACCCCGTGCTGTGCAATGTGGAAACAATCCAGTGGCGGGACATCGTG<br>GACAACGACTTCATCAGCAACATGAGCATGGACATCCAGAACCAGGCCGGCAGAT<br>GCCAGAAGTGCGACCCCAGCTGTCCCAACGGCTCTTGTTGGGGCCCTGGCAAAGA<br>GAACTGCCAGAAGCTGACCAAGATCATCTGCGCCCAGCAGTGTAGCGGCCGGTGC<br>AGAGGCAGAAGCCCTAGCGACTGCTGCCACAACCAGTGTGCCGCCGGATGTACCG<br>GCCCCAGAGAAAGCGATTGCCTCGTGTGCCGGAAGTTCCGGGACGAGGCCACATG<br>CAAGGATACCTGCCCCCCTCTGATGCTGTACAACCCCACCACCTACCAGATGGAC<br>GTGAACCCCGAGGGCAAGTACAGCTTCGGCGCCACCTGTGTGAAGAAGTGCCCCA<br>GAAACTACGTCGTGACCGACCACGGCAGCTGCGTGCGGGCCTGTAGCAGCGATAG<br>CTACGAGGTGGAAGAGGACGGCGTGCGGAAGTGCAAGAAATGCGAGGGCCCCTGC<br>CGGAAAGTGTGCAACGGCATCGGCATCGGAGAGTTCAAGGACACCCTGAGCATCA<br>ACGCCACCAACATCAAGCACTTCAAGAACTGCACCAGCATCAGCGGCGACCTGCA<br>CATCCTGCCCGTGGCCTTTAGAGGCGACAGCTTCACCCACACCCTGCCCCTGGAC<br>CCCAAAGAGCTGGACATTCTGAAAACCGTGAAAGAGATTACCGGCTTCCTGCTGA<br>TTCAGGCCTGGCCCGAGAACCGGACAGACCTGCACGCCTTCGAGAATCTGGAAAT<br>TATCAGAGGCCGGACCAAGCAGCACGGCCAGTTTTCTCTGGCCGTCGTGGGCCTG<br>AACATCACCAGCCTGGGCCTGCGGAGCCTGAAAGAAATCAGCGACGGCGACGTGA<br>TCATCTCCGGCAACCGGAAGCTGTGCTACGCCAACACCATCAACTGGAAGAAGCT<br>GTTCGGCACCAGCTCCCAGAAAACAAAGATCATCAACAACAAGGACGAGAAGGCC<br>TGCAAGGCCATCGGCCACGTGTGCCACCCTCTGTGTAGCTCCGAGGGCTGTTGGG<br>GACCCGGCCCTAGAGATTGCGTGTCCTGCAGAAACGTGTCCCGGGGCAAAGAATG<br>TGTGGAAAAGTGCAACATCCTGGAAGGCGAGCCCCGCGAGTTCGTGGAAAACAGC<br>GAGTGCATCCAGTGCCACCCCGAGTGTCTGCCCCAGGCCATGAACATTACCTGTA<br>CCGGCAGAGGCCCCGACAGCTGCATCAAGTGCGCCCACTACATCGACGGCCCCCA<br>CTGCGTGAAAACCTGTCCTGCCGGCATCATGGGCGAGAACAACACCCTCGTGTGG<br>AAGTTCTCCGACGGCAGCCGGATGTGTCACCTGTGCCATCCCAACTGCACCTACG<br>GCTGTGAAGGCCCCGGACTGGAAGGCTGTGCCAAGCCTGGACCCAAGATCCCCAG<br>C**CACCACCACCATCACCAC****TGA**

Color coding: green – leader sequence of human EGFR; black – coding sequence for expression of soluble canine EGFR; blue – 6x HIS-tag; red – stop codon

Supplementary Table 2: Patients' characteristics of serum stability samples

Patient #	Breed	Age	Sex	Disease	ICD-10	Specific tumor type/ histopathology	Grade	Stage
**1**	german pinscher	11y7m	f, neutered	mammary carcinoma	C 80	complex tubulopapillary carcinoma	I	I
**2**	mixed breed	11y7m	f, neutered	mammary carcinoma	C 80	simple solide carcinoma	II	II
**3**	cocker spaniel	10y	f	mammary carcinoma	C 80	simple tubulopapillary carcinoma	I	I
**4**	border collie	n.a.	f	mammary carcinoma	C 80	simple tubulopapillary carcinoma	I	II

Y – year, m – month, f – female, n.a. – no data available

Supplementary Table 3: Patients' characteristics of autoradiography sections

Sample letter	Breed	Age	Sex	Disease	ICD-10	Specific tumor type/ histopathology	Grade	Stage
**A**	German shepherd	12y6m	f, neutered	mammary carcinoma	C 80	solid to tubular	n.a.	n.a.
**B**	poodle	9y11m	f, neutered	mammary carcinoma	C 80	tumor with focal cystic cavities	n.a.	n.a.
**C**	border terrier	11y3m	f	mammary carcinoma	C 80	scirrhous	n.a.	n.a.

Y – year, m – month, f – female, n.a. – no data available
